# Stem cell biology needs a theory

**DOI:** 10.1016/j.stemcr.2022.11.005

**Published:** 2023-01-10

**Authors:** Ben D. MacArthur

**Affiliations:** 1 Alan Turing Institute, London, UK; 2 Mathematical Sciences, University of Southampton, Southampton, UK; 3 Faculty of Medicine, University of Southampton, Southampton, UK

## Abstract

Stem cell biologists are increasingly making use of computational models to decipher their data. However, there is sometimes uncertainty about what makes a “good” model. The purpose of this commentary is to argue for closer integration of experiment and theory in stem cell research and propose guidelines for good theory.

## Introduction

Recent years have seen the development of experimental methods that are able to collect extraordinary amounts of data concerning stem cell identity, fate, and function. While undoubtedly transformative, these advances also present us with a challenge: how do we make best use of the resulting data? To approach this challenge, many researchers are turning to advanced mathematical, computational, and machine-learning methods.

When doing so, there is often an implicit belief that if we could just “let the data speak”—i.e., if we collect as much data as possible and mine it in the right way—then insight will naturally emerge. This belief, which might be called the fundamental dogma of big data, has arguably been very successful. For instance, unsupervised mining of large repositories of single-cell data, such as the various cell atlases, are revealing ever richer cellular ecologies, allowing us to dissect the molecular basis of cellular functions in ever greater detail and transfer insight between organisms (Elmentaite et al., 2022).

Yet, because data collection and analysis are not value free, it can also be misleading. When we collect data via experiments, we make numerous choices—which variables to measure and which to exclude, which analytical tools to employ and to what purpose, etc.—that reflect prior knowledge and beliefs and judgments of what is important. As the philosopher of science Mary Midgely has noted, “Facts are not gathered in a vacuum, but to fill gaps in a world-picture which already exists” (Midgely, 2002). Thus, data alone do not deliver insight in the same way that an experiment alone—without interpretation, contextualization, and harmonization with other sources of information and the wealth of pre-existing knowledge—does not deliver insight. Data cannot speak. It is data interpreted in the light of theory that advance scientific understanding.

In the context of stem cell biology, two issues are particularly relevant. First, “world pictures” or theories can be carried unconsciously, and so can be hard to articulate, and may therefore inform experiments in unappreciated ways. For example, in the case of clustering of single-cell sequencing data, a common assumption is that compact clusters in “expression space” map to discrete functional identities in “phenotypic space” (Casey et al., 2020). This seems like a natural assumption to make and is undoubtedly helpful in making sense of complex data. But why should it be true? It is not *a priori* obvious that the mapping between genotype and phenotype should be so well structured. Different patterns of gene and protein expression could, for instance, map to the same cellular function under different circumstances; similarly, the same patterns of expression could map to distinct functions under different conditions. In both these cases, it would not be apparent what any identified clusters mean, if anything, in terms of cell function. Yet, this process is remarkably successful, and identified clusters often do map to defined cell identities, suggesting that it is consistent with some deeper principles of cell biology rather than pure contingency. One now well-established theory is that distinct cell functions are associated with “attractors” (essentially preferred, balanced, states) of complex underlying dynamical systems, which are governed by networks of interactions between genes and their products and environmental regulators (Huang et al., 2005). Thus, compact patterns of gene and protein expression relate to distinct cellular functions because underlying dynamical constraints force structure to the mapping between genotype and phenotype, which often, although not always, manifests as clusters in high-throughput single-cell expression data. The process of unsupervised clustering works well, then, because it is underpinned by a prototypical theory and its success, while not definitive proof, is evidence of some truth in the underpinning theory.

Second, the insights that data provide are typically purely descriptive or associative, while the fundamental questions of science are causal. Thus, while we may be able to identify distinct cell populations from large single-cell sequencing databases via unsupervised clustering methods, we may still have little understanding of *why* these particular clusters emerged or their broader biological significance. Indeed, although such taxonomies can provide a useful way to organize or compress complex experimental datasets, they cannot provide answers to important “why” questions (why do cells arrange themselves in this way and not that? Why do these genes conspire with those environmental effects to produce that phenotype and not another?). Answers to such why questions are not accessible from data alone and cannot properly be obtained without a framing worldview—even if it is only partial—within which to interpret the data. As Judea Pearl, the father of causal inference, has said, “Causal questions can never be answered from data alone. They require us to formulate a model of the process that generates the data …” (Pearl and Mackenzie, 2019). In other words, they require a theory.

## What makes a good theory?

In the mathematical and physical sciences, theories are often presented in terms of mathematical equations (known as mathematical models or, if simulated on a computer, computational models), although they do not need to be. A theory is simply a set of statements or principles that explain a set of data—Darwin’s principles of evolution by natural selection, for example, are a theory in prose, rather than mathematical, form. With this in mind, we might ask: what makes a good theory in stem cell biology? As a start, I suggest the following five principles.

First, a good theory should be as simple as possible but no simpler (Robinson, 2018). It should include everything that is necessary and nothing that is not. It does not have to be perfect or irrefutable, but it should be necessary and sufficient to explain the data at hand and be both clear and clarifying. A good example of an elegant theory that explained the data of its time is Till and McCulloch’s classic stochastic model of stem cell proliferation (Till et al., 1964).

Second, it should have explanatory power. A theory is powerful if it can economically explain a large range of data. However, it should not explain everything. A theory that can explain any conceivable data does not have discriminatory power and so is not useful. Rather, it should make precise predictions that are experimentally falsifiable. The final verdict on a theory’s utility must be delivered by experiment, and so the predictions of a good theory must be amenable to experimental verification, at least in principle. Waddington’s epigenetic landscape is a good example of a powerful theory that is nonetheless circumscribed in its utility (Waddington, 1957).

Third, it should be practical and guide better experiments. Theories that are only useful in theory are not useful. The predictions of a theory may, of course, be beyond the reach of current experimental technologies. But, by defining precise and realistically testable hypothesis, a good theory can stimulate the development of new experimental approaches and new ways of combining experimental methods. The classical Luria-Delbrück fractionation experiments are a good example of theory guiding the design of a powerful experiment (Luria and Delbrück, 1943).

Fourth, it should bring coherence. By providing a new way to look at the world, a good theory can harmonize apparently contradictory or conflicting sources of evidence and stimulate new ways of looking at old data. A good recent example of this is provided by Greulich and colleagues, who show how universal stem cell properties and principles of cell lineage architectures emerge naturally from theoretical consideration of the dynamics of homeostasis in renewing tissues (Greulich et al., 2021).

Fifth, it should generalize from the particular to the universal. A good theory should be able to place specific biological facts within their wider context and identify general principles that lead to similar observations in different situations. A good recent example is the work of Sáez and colleagues, who show, via elegant mathematical reasoning integrated with experiment, how cell-fate decisions can be grouped into universality classes governed by common geometric principles (Sáez et al., 2022).

## Conclusion

In this commentary, I have argued that theory is not a dispensable add on to the main business of experimental science but rather can—and should—play a central part. Good theory is essential to making best use of data and advancing understanding. Moreover, theory and experiment are not independent of each other: as an ideal, they should enhance each other by a process of “adversarial collaboration” (Cleeremans, 2022) in which theory forces experimental innovation and experiments seek to falsify theories. Theory should advance experiment; experiment should improve theory.

Developing a closer relationship between theory and experiment will require a change in mindset, however. Because theory and experiment are not two distinct domains, we should not see experiments simply as a source of data or theory simply as a source of abstract knowledge. Rather, they should work together toward the common goal of generating practical new ideas about the natural world (Nurse, 2021). While tremendous progress continues to be made in experimental stem cell biology, progress in theory is lagging—some beautiful examples notwithstanding (Clayton et al., 2007). We should work to rectify this by drawing on tools from across the mathematical, physical, and computational sciences. Doing so will allow us to develop a deeper understanding of the universal principles of stem cell fate and function and a firmer grasp of the rich cellular and molecular specifics of their regulation.
